# Moment-to-Moment Continuous Attention Fluctuation Monitoring through Consumer-Grade EEG Device

**DOI:** 10.3390/s21103419

**Published:** 2021-05-14

**Authors:** Shan Zhang, Zihan Yan, Shardul Sapkota, Shengdong Zhao, Wei Tsang Ooi

**Affiliations:** 1 NUS-HCI Lab, Department of Computer Science, School of Computing, National University of Singapore, Singapore 117417, Singapore; zihanyan@zju.edu.cn (Z.Y.); shardul@u.yale-nus.edu.sg (S.S.); zhaosd@comp.nus.edu.sg (S.Z.); 2 College of Computer Science and Technology, Zhejiang University, Hangzhou 310058, China; 3 Division of Science, Yale-NUS College, Singapore 138527, Singapore; 4 National University of Singapore, Singapore 117417, Singapore; ooiwt@comp.nus.edu.sg

**Keywords:** EEG, moment-to-moment, attention detection, wearable, machine learning

## Abstract

While numerous studies have explored using various sensing techniques to measure attention states, moment-to-moment attention fluctuation measurement is unavailable. To bridge this gap, we applied a novel paradigm in psychology, the gradual-onset continuous performance task (gradCPT), to collect the ground truth of attention states. GradCPT allows for the precise labeling of attention fluctuation on an 800 ms time scale. We then developed a new technique for measuring continuous attention fluctuation, based on a machine learning approach that uses the spectral properties of EEG signals as the main features. We demonstrated that, even using a consumer grade EEG device, the detection accuracy of moment-to-moment attention fluctuations was 73.49%. Next, we empirically validated our technique in a video learning scenario and found that our technique match with the classification obtained through thought probes, with an average F1 score of 0.77. Our results suggest the effectiveness of using gradCPT as a ground truth labeling method and the feasibility of using consumer-grade EEG devices for continuous attention fluctuation detection.

## 1. Introduction

Attention is a neurocognitive process critical to a wide variety of everyday tasks [1]. Maintaining one’s attention for a period of time, and selectively concentrating on a stimulus or task while ignoring others require effort, and vary based on the individual’s ability to withstand cognitive load [2]. With the rising scale of distractions presented as part of modern living, several research works dived into building interfaces or systems to facilitate attention processes, e.g., attentional user interface [3], attention-aware systems [4] and attention management systems [5]. The fundamental step in building these systems is measuring users’ attention states.

However, due to the lack of a reliable method of labeling attention states, moment-to-moment attention fluctuation measurement is unavailable in the current attention-related research in the computer science (CS) communities.

Currently, discrete data collection methods, such as thought probes, self-report questionnaires and surveys [6,7,8,9], have most often been used to establish an individual baseline for attention states. However, these methods only collect discrete data, i.e., the attention states at the moment, and cannot reflect the continuous nature of attention [10] as data concerning the changes from the start to the end of the attention states are unavailable. Researchers have also used controlled tasks to label users’ attention states over the time period of the task. For example, experiments on divided attention [11] are designed such that full attention states are labeled as learning without distractions, and external distraction states are labeled as learning under environmental noise. It is, in reality, unlikely that users remain fully in the respective states throughout their tasks. In a nutshell, current labeling methods for attention states offer limited options for data collection; data on attention are either collected in the form of discrete measurements (“moments in time”), or in the form of summaries over a long period of time [12]. However, sensing continuous and dynamic attention requires accurate labeling on attention with a regular frequency over a period of time, and current methods, therefore, do not offer the complete picture for understanding attention.

On the other hand, psychologists have explored methods such as vigilance tests to label attention states based on participants’ behavior performance. Traditional attention-measuring tests include Mackworth’s clock test [13], A-X Continuous performance tests (A-X CPTs) [14], and Sustained Attention to Response Task (SART) [15] etc., but they all have their own limitations [2]. In Mackworth’s clock test, subjects observed the pointer moving around a clock for up to 2 hours and were instructed to press a key when they observed an infrequent double moving of the pointer. In A-X CPTs, subjects were shown a series of letters and asked to respond when they noticed an “X” or an “X” appearing after an “A”. In SART, subjects responded to the majority of stimuli and withheld responses to rare targets [15,16]. These tests are limited, as they merely observe the behavioral analysis of error rate (ER) and response time (RT), i.e., they relate low attention states to higher ER and slower RT. However, ER and RT do not always account for high frequency attention fluctuation [17,18,19], as extremely fast RT could stem from inattention [20]. Recent neurophysiological studies have highlighted the use of response time variability (RTV) as a more effective characterization of neural networks supporting attention [2]. The second limitation is associated with test design; when image stimuli are abruptly presented, they become alert cues that affect participant attention levels [2,21].

Building on these methods, Rosenberg et al. [22] proposed the gradual-onset CPT (gradCPT). The gradCPT adds to its predecessors in two major ways: firstly, gradCPT analyzed participants’ RTV, which is more precise and enables the continuous measurement of high-frequency attention fluctuation [17,23,24]; secondly, the gradCPT introduced a gradual visual onset (800 ms), thus minimizing unintended effects from sudden stimuli onset. Hence, we choose the gradCPT as our ground truth labeling task. GradCPT [22] divided attention into attentive “in the zone’’ and not attentive “out of the zone’’ states. GradCPT not only allows for more precise attention labeling, but also increases the time resolution of attention-state labeling to the sub-second level (800 ms). In addition, we used a commercial EEG device to collect data on 18 participants and trained a support vector machine (SVM) on attention state classification in a user-dependent manner, with an average accuracy of 73.49%. The results showed a solid step forward towards the ultimate goal of supporting continuous, fine-grained measurement of attention state using relatively inexpensive and accessible sensing devices, and a significant increase in accuracy in predicting attention states, as compared with a recent EEG-based prediction on mind-wandering [25], which leveraged participants’ probe responses during sustained-attention-to-response task (SART) and showed an average accuracy of 60% in mind-wandering detection across tasks.

To validate the effectiveness of our EEG-based classification method, we tested it in a video learning scenario for the ease of comparison with the rich work in attention detection in this scenario. We found that our EEG classified attention fluctuation labels match with classification obtained through thought probes with an average F1 of 0.77, which is comparable to that achieved by recent investigations that employ discrete sampling techniques [26,27]. This study fills the gap in existing research by achieving reasonable accuracy for continuous attention measurement with the sub-second scale. Our two studies demonstrated the effectiveness of using gradCPT as a ground truth labeling method and the feasibility of using EEG for continuous attention fluctuation detection across tasks on a sub-second scale.

The contribution of this paper is three-fold:

1. We applied gradCPT, a well-established method in psychological research, to collect continuous attention fluctuation labels. Based on gradCPT, we developed a new technique for measuring continuous attention with consumer grade EEG devices and achieved 73.49% of accuracy in detection of attention fluctuation for the sub-second scale, moment-to-moment.
2. We empirically validated our technique in a video learning scenario, which suggested the feasibility of predicting learners’ continuous attention fluctuation while watching lecture videos.
3. We discussed both research and application implications of measuring continuous attention fluctuation using EEG for future studies.

## 2. Related Work

### 2.1. Attention State Classification

In this section, we discuss existing technology-based approaches for measuring attention states. In general, data regarding attention state transition are collected via behavioral or physiological measurements. Previous research has explored behavioral cues, such as head pose, body posture [28,29]), facial expression [30] and reading behaviors, such as reading time on a given paragraph [31]. For instance, Zaletelj et al. [32] classified high, medium, and low states of attention through Kinect features with 75% accuracy. Wang et al. [28] divided attention states based on eye closure. However, it is difficult to establish a uniform and consistent approach to measure attention due to contextual and individual differences. Attention state fluctuations (e.g., mind wandering) may also be imperceptible as behavioral cues [33].

An alternate line of research on attention state classification has investigated the use of physiological sensors to measure attention states (see a summary of recent works in Table 1). For example, Abdelrahman et al. [12] used thermal imaging and eye-tracking to classify four types of attention states based on the clinical model. Di et al. [34] used electrodermal activity (EDA) to classify ‘engaged’ and ‘not engaged’ attention states of students during lectures with a 60% accuracy level. Xiao et al. [11] explored the use of fingertip photoplethysmogram(PPG) signals to classify four levels of divided attention. These works, however, relied on discrete sampling or designed experiment conditions as ground truth, and classified attention states over longer time spans of several minutes, which suggests that their measurements techniques are unsuitable for fine-grained attention measurement.

### 2.2. EEG-Based Attention Research

Due to the close link between brain neural networks and attention, and EEG being recognized as “a genuine window on the mind” [35], researchers in neuropsychology have explored the correlations between EEG changes and cognitive activities in different ways [36,37,38]. For instance, Ko et al. [36] demonstrated the relationship between variations in EEG spectral dynamics and prolongation of the RT for the sustained attention task in real classroom settings; Behzadnia et al. [37] investigated the changes in EEG frequency bands using a conjunctive continuous performance task. Zeid et al. [38] showed the changes in spectral behavior and event-related potential of EEG in 5 min awake and drowsy states.

These works demonstrated the feasibility of using EEG to measure attention and led researchers to further explore the EEG-based classification of attention states. For instance, Wang et al. [39] used EEG signals collected from a 32-electrode device to classify attention focus in the driving context and achieved an accuracy of around 85%. Attention was labeled through RT analysis on math problems which appeared at 6–8 s intervals. Vortmann et al. [40] used EEG signals collected from a 16-electrode device to classify internally and externally oriented attention states in the augmented reality setting, and achieved an accuracy of 85.37% in a 13-s time scale. Di et al. [41] developed an adaptive automation system with EEG signals from a 15-electrode device and eye-tracking technique. The vigilance level was measured in 5 min intervals. However, these studies are limited in that they focused on specific aspects of attention under unique contexts, the results of which may not be generalized to other contexts. In addition, relatively complex EEG devices were used, making it difficult to replicate in a real world context.

A few researchers trained their EEG data based on psychology tests and applied it in real-world scenarios. Jin et al. [25] applied a SART to predict mind wandering using EEG and achieved an average accuracy of 60%. Chen et al. [42] used an A-X continuous performance test (CPT) to classify attention states using EEG. Sebastiani et al. [43] identified EEG features that related to decreases in vigilance on the minute time scale. However, the tests they selected are based on ER or RT analysis, whereas the state-of-the-art psychology tests utilized RTV to label attention states, which was shown to be a more accurate behavior marker for attention state changes [2]. Furthermore, these tasks were unable to reveal continuous fluctuations in attention. In this paper, we applied the most recent RTV analysis-based attention research in psychology, the gradCPT [44], to the application level of attention measurement. The gradCPT allows for precise attention labeling on an 800ms time scale, and its divided attention states could reflect brain network activity, thereby accounting for the fundamental mechanism of attention control, increasing its generalizability and applicability to different contexts. To our knowledge, our research pioneers the use of gradCPT as ground truth labeling and the use of EEG signals (obtained from a portable EEG device) to effectively measure continuous attention fluctuations on a sub-second scale.

In the following sections, we present our data collection and model training methods, along with descriptions of the gradCPT task and devices used. In addition, we detail our pre-processing, feature extraction and classification approaches, and report the results. Next, we test our classification algorithm in a video learning scenario to verify that the EEG-based model applies in the wild. Lastly, we discuss how the findings of our work can be applied to future studies.

## 3. Methods

We first collected a dataset of EEG signals from 18 participants who completed three sessions of gradCPT, then built a classifier on attention fluctuation prediction based on EEG data. We describe the gradCPT, experiment setup, procedures, preprocessing and model training in the following sections.

### 3.1. Experiment

#### 3.1.1. Attention Labeling by the gradCPT

**Figure 1:**
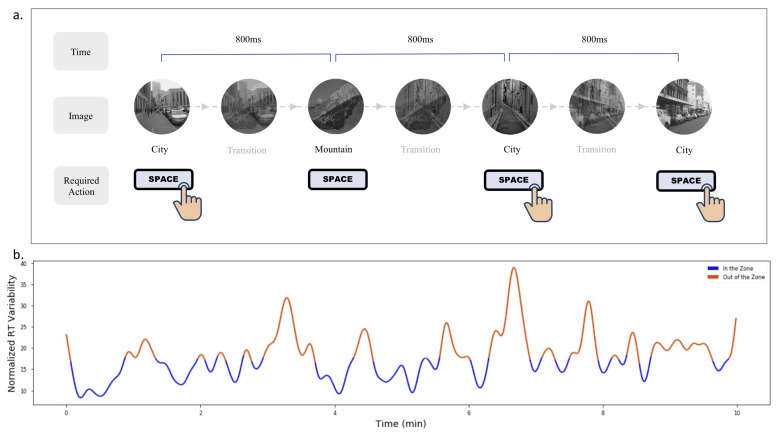
(**a**) An illustration of three continuous trials of the gradCPT. (**b**) A division of “in the zone” and “out of the zone” states based on the participant’s RTV over 10 min.

We use the original design of gradCPT introduced by Esterman et al. [44]. GradCPT’s stimuli comprises 20 images, in which 10 feature city scenes, and the other 10 feature mountain scenes. The city images are used as target images, appearing 90% of the time, and the mountain images are used as non-target images, appearing 10% of the time. A notable design feature in gradCPT involves the gradual transition of one image stimuli to another, where the opacity transition is 800 ms. Participants are instructed to press a key (space bar) when they notice the city scene, and withhold responses when mountain scenes appear (Figure 1a). The gradCPT implementation calculates RT relative to the beginning of each image transition. So an RT of 800 ms indicates that a user pressed the button when the image was 100% coherent and not overlapping with other images. Esterman et al. [44] further detail how the gradCPT, in cases of multiple button presses, can disambiguate the responses.

The RTV, which refers to the trial-to-trial variation in RT, was calculated to classify attention states. In gradCPT, the within subject analysis for RTV is computed via the variance time course (VTC) on the z-normalized RTs. The VTC value represents the absolute deviation of the trial’s RT to the mean RT of the entire run. The missing values for trials without responses are linearly interpolated, as described by Esterman et al. [44], from the RTs of the two surrounding trials. This VTC is further smoothened by using a Gaussian kernel of nine trials (7 s) full-width at half-maximum (FWHM). Given that the VTC is initially computed by taking the absolute difference of the RT at a trial with the mean RT of the entire run, extremely fast or extremely slow RT will have higher absolute value of the variation. This allows the values of the VTC higher than the median to capture these extreme responses and mark them as “out of the zone” attentional states.

We use the original Matlab script developed in Esterman’s experiment [44], with a slight modification to include timestamps for the start and end of each session for time synchronization. The script calls functions from the Psychophysics Toolbox79 within Matlab R2020b to construct the environment. The script is run on a desktop machine (16 GB RAM, Intel 7 processor) with the Windows 10 operating system, and has a 23-inch (1920 × 1080 resolution) monitor with a refresh rate of 60 Hz.

#### 3.1.2. Setup

**Figure 2:**
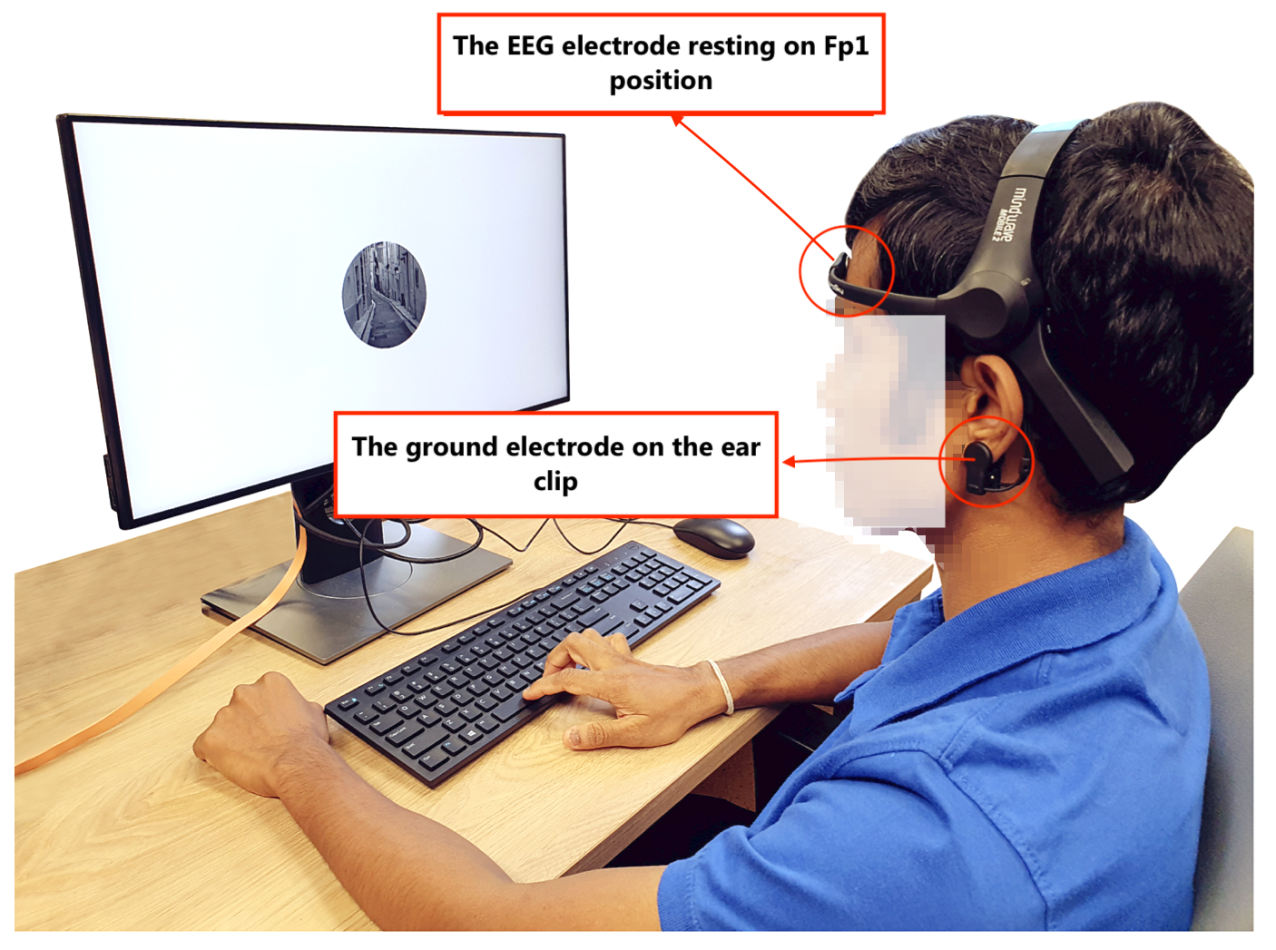
The experiment setup—a participant wearing the EEG device, engaged in gradCPT.

Figure 2 illustrates our experimental setup. We used a popular portable EEG device, Neurosky Mindwave mobile 2, with a dry electrode placed on the forehead above the left eye (pre-frontal left position, Fp1 in the 10–20 system) and a ground electrode on the ear clip. The sampling rate is 512 Hz. The EEG readings from the sensor were sent via Bluetooth to the server on a desktop. We choose the device with the measuring electrode on Fp1 for two reasons: (1) this position is free of hair, which allows for easy placement; (2) the frontal lobe is the part of the brain that controls important cognitive activities such as attention, which makes it easy to capture brain signals that are related to attention [45]. Compared with traditional wet-electrode EEG devices, which can be cumbersome, this device is easy to wear and is low-cost.

The EEG device supports two output formats: the raw EEG signal generated from Fp1, and the derived index of attention and meditation generated from the built-in algorithm of Neurosky. While the latter was used in some research works [46], conflicting results on the validity of derived attention index were reported [47,48,49]. The concern of the raw signal focused on the influence of eye blinks on the raw EEG signal recording [50]. In this paper, we only used the raw EEG signal, as it has been validated by many research works [48,51,52,53]. Besides using the Neurosky device’s original noise-removal algorithm, we also introduced our own eye-blink detection (Section 3.2.2) and noise-removal algorithm to minimize the influence of eye blinks on EEG readings.

#### 3.1.3. Participants and Procedure

In total, 18 participants (8 females, $age_{M}$ = 23.94, $age_{SD}$ = 3.17) were recruited through university mailing lists. The research was reviewed and approved by the Departmental Ethics Review Committee of the local university. All participants were right-handed, with normal or corrected-to-normal vision.

Upon arrival, participants were briefed about the experimental procedure and signed the consent forms. They were asked to wear the sanitized EEG sensors to their forehead and left earlobes, as shown in Figure 2. Once the sensors were properly worn, participants were asked to ignore the sensor during the experiment. They were also told to avoid head, jaw-teeth, and mouth movements, such as mouth-clenching, as far as possible, as they can limit the influence of muscle activities on the EEG signal. Participants were then instructed to perform a 1 min practice session of gradCPT to familiarize themselves with the setup. After the practice session, they were asked to perform three runs of gradCPT, each run lasting 10 min, with a 2-min break in between each. We ran three 10-min sessions so as to collect more data for machine learning training while reducing the possibility of fatiguing the participants. Ten minutes is the typical duration for a gradCPT session [22], and having multiple runs of 10-minute sessions is common in previous studies to collect more data [54,55]. The entire experiment lasted around 45 min and each participant received approximately USD 7.40 for their time.

### 3.2. Preprocessing

Besides brain electrical activity, EEG electrodes can record environmental electromagnetic interference or eye-blink artifacts [56,57]. Following the EEG preprocessing steps in [58,59], we applied normalization, artifacts removal and bandpass filtering on the extracted EEG signals.

#### 3.2.1. Normalization

**Figure 3:**
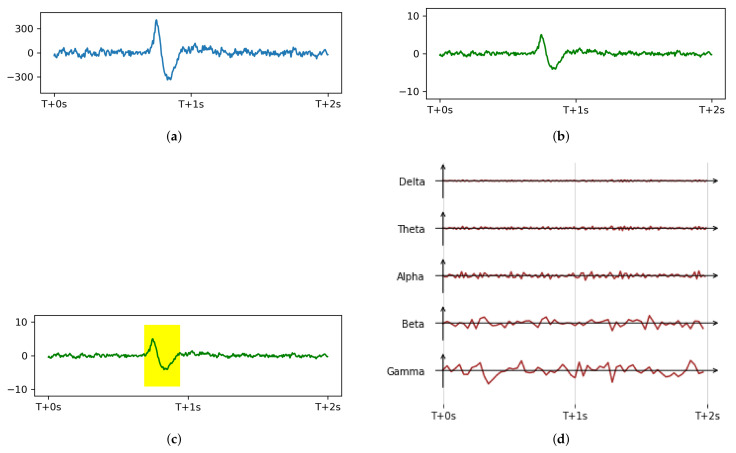
Preprocessing procedure (**a**) a 2 s raw EEG signal segment example where the participant had a natural blink; (**b**) normalized signal; (**c**) EOG detection on the normalized signal; (**d**) five frequency bands after bandpass filtering.

Due to individual differences among participants, the resulting data distributions have distinct differences that make the model difficult to train. In order to remove personal factors unrelated to the task, which may otherwise affect the training model, we followed Zhu et al. [60] and Zhang et al. [61] and normalized data from each individual using z-score normalization. Figure 3 shows the raw signal and signal after normalization Figure 3b.

#### 3.2.2. Artifacts Removal

EEG is susceptible to both extrinsic artifacts from external environment and intrinsic artifacts from physiological activities of the body [62]. The main extrinsic artifact is the power line artifact, which is removed through a 50 Hz notch filter. Among intrinsic artifacts, eye blink represented by EOG (electrooculogram) and muscle activity represented by EMG (electromyogram) are the two artifacts that influence EEG. The frequency range of these two artifacts overlap with EEG signals, and thus cannot be removed through bandpass filtering [62].

Previous studies have relied on the Neurosky device’s built-in noise removal algorithm [42,63], though we have noticed that, in practice, eye blinks (EOG signals) significantly influence EEG signals (Figure 3). Until now, noise removal methods for single channel EEG devices are relatively limited. Linear regression [64,65] and adaptive filtering, for example, require additional reference channels with the artifacts waveforms [66]. Blind source separation methods decompose EEG signals into components and then reconstruct, but also require a large number of electrodes [67]. The most recent work uses deep learning networks [68], but has a high requirement on the size of the dataset, and the technical cost is high, too. Thus, we used a threshold value to detect EOG amplitude peaks in the EEG signal, then a morphological method to remove false-positives and negatives to mark a continuous EOG range (Figure 3c). According to our experiment data, the EOG effects lasted a maximum of 450 ms. After the EOG artifacts had been identified, we replaced the features of the EOG artifact area with corresponding values of the previous area (without EOG artifact) in the same duration.

While feature replacement could introduce bias, as the resolution of the attention label is 0.8 s, the effect of bias from the replacement of 450ms EOG signal could be limited and not cause significant errors that could change the results. Erasing the epochs containing EOG completely would result in the unavailability and the waste of a considerable amount of data. In addition, to address the problem of independence in the time series signal, when extracting the features for the classifier, we include features from the nine preceding sliding windows to capture the effect of trial at time t on subsequent trials. The selection of nine preceding windows was based on the original gradCPT study of Esterman et al. [44], which used a Gaussian smoothing kernel of nine trials, full width at half maximum, to compute the VTC value for a given trial.

#### 3.2.3. Bandpass Filtering

The EEG frequency bands include delta (0.5–4 Hz), theta (4–8 Hz), alpha (8–12 Hz), beta (13–30 Hz), and gamma (>30 Hz) waves. Thus, we applied a 0.5–50 Hz bandpass filter to remove electromagnetic interference, while retaining the common EEG frequency bands. The signal after bandpass filtering can be seen in Figure 3d.

### 3.3. Feature Extraction

**Figure 4:**
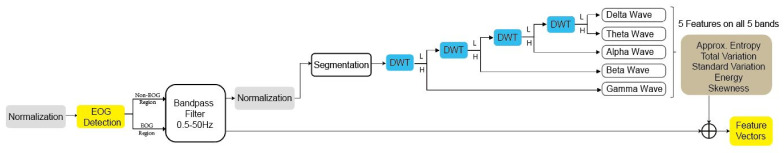
Model of classification.

**Figure 5:**
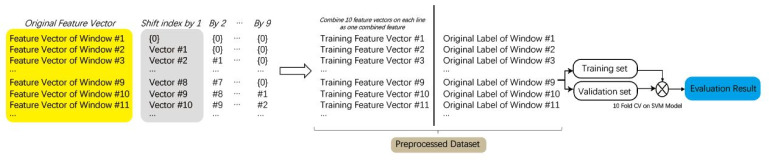
Feature extraction process.

The model of classification can be seen in Figure 4. We segmented the EEG signal into non-overlapping 0.8 s segments with a corresponding gradCPT label (1 for “in the zone” and 0 for “out of the zone”). For each participant, 2250 labels were collected for the label with 800 ms interval in 30 min. To extract the frequency features related to each frequency band, we used the discrete wavelet transform (DWT) for multilevel decomposition to separate signals from five different EEG frequency bands. We computed five features (approximate entropy, total variation, skewness, standard deviation, and energy) for each band. The feature descriptions can be seen in Table 2. The feature vectors of three sessions for each participant were collected from the z-normalized signal so that they are scale- and offset-invariant. To address the problem of independence in the time series signal, after extracting the features, we added features from nine preceding sliding windows as the features of this window (for the first nine windows, we used none to fill non-exist values) to get 250 features in each sliding window for training (Figure 5). The nine preceding windows was based on the original gradCPT study of Esterman et al. [44], which used a Gaussian smoothing kernel of nine trials, full width at half maximum, to compute the VTC value for a given trial. This could enable the model to better learn the temporal features.

### 3.4. Classifier

There are numerous machine learning classifiers [69] such as k-nearest neighbor (kNN), SVM, Bayesian, as well as deep learning networks [70], such as the convolutional neural network (CNN) and long short-term memory (LSTM), though we chose the SVM with the radial basis function kernel, due to its popular use and in consideration of our relatively small sample data size for each participant (2250 samples). To classify each 0.8 s EEG segment as “in the zone” or “out of the zone”, a different SVM model was trained for each participant. To avoid overfitting the model, a 10-fold nested stratified cross-validation (CV) was used to estimate the model’s generalization performance. The Nested CV implementation was based on recent research by Vabalas et al. [71], who showed that biased performance estimates arise from regular CV, but can be avoided with Nested CV, regardless of the sample size. Stratified sampling was used to ensure that the percentage of samples of the two classes in each fold was approximately the same as that of the entire data set. To note, not all features in the dataset are used to train the model. We use Student’s t-test to assess and pick the ten most relevant features and train and evaluate the model using the ten feature we selected in each fold. The final model’s performance was taken from the average of the balanced accuracy in the classification on the validating set in each fold.

## 4. Classification Result and Discussion

**Figure 6:**
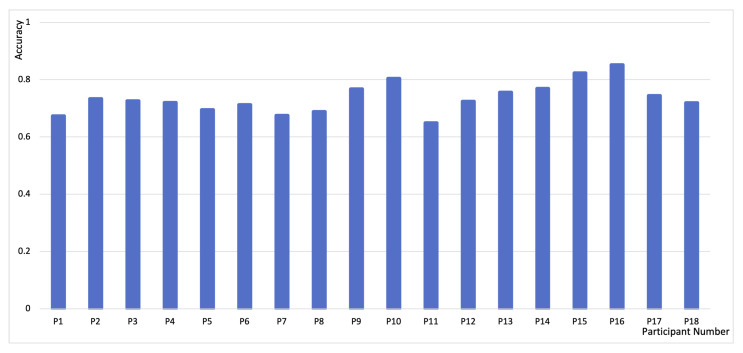
Accuracy of 18 participants, P1–P18.

Figure 6 shows the performance obtained using the trained classifier and applying the 10-fold Nested CV protocol described in Section 3.4. Overall, the model achieved an accuracy of 73.49% (SD = 0.05). The average F1 is 0.76 (SD = 0.06). While this accuracy does not seem high, it is significant, considering the sub-second time scale, generalizability of the task, and application potential of the commercial grade, low-cost EEG device. A state-of-the-art mind-wandering EEG prediction by Jin et al. [25] leveraged participants’ probe responses during SART and showed an average accuracy of only 60% in mind-wandering detection across tasks. Our work demonstrates the feasibility of measuring continuous attention fluctuation in a sub-second time scale, using commercial EEG devices.

**Figure 7:**
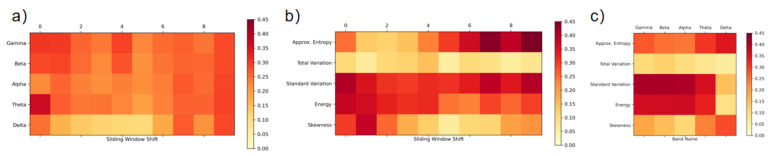
Mean correlation coefficient for different (**a**) frequency band and sliding window shift; (**b**) feature type and sliding window shift; (**c**) feature type and frequency band.

To present the importance result of each feature, we calculated the mean correlated coefficient for different participants, between the attention label and all 250 features separately. Since the 250 feature input for each window can be organized into five different feature types on five different bands for 10 different adjacent sliding windows, we can assess the result in three different perspective. We averaged the result of 10 sliding window shifts and presented the mean correlated coefficient of 25 features as a colored matrix (Figure 7c). The color of each cell indicates the average correlation coefficient; the darker the cell, the more important the feature. Similarly, we can average the result of five feature types (Figure 7a) or five bands (Figure 7b) to reveal the mean correlated coefficient of bands or feature types on different sliding windows (describe as sliding window shift).

Even though this is a user-dependent model, by averaging the result for each participant, we can conclude from Figure 7c that the eight combinations of energy, standard variation of alpha, beta, gamma and theta bands, stand out as dominant features, followed by approximate entropy on delta and theta bands.

## 5. Validation Study—Detecting Attention Fluctuation in Video Learning

In the previous section, we demonstrated the feasibility of our technique in classifying continuous attention fluctuation on an 800 ms time scale. To validate its usability in the real-world, we tested the performance of our technique for predicting learner’s attention fluctuation using a video learning scenario. We chose a video learning scenario because of (1) the ease of implementation and testing, and (2) the rich and comparable body of work on attention detection in this scenario. Attention measurement in the video learning scenario is a popular research topic, given the strong correlation between attention with learning performance [72]. We expect that our technique’s predictions can match video-learners’ attention states.

Our first step was to label the learner’s attention states. While it may be obvious that high attention levels indicate high learning performance, learning performance tests are not often used to assess learners’ attention states because attention is merely one cognitive facility amongst many others utilized during test taking. Instead, thought probes and self-reports [73] are more commonly used to assess learners’ attention states. With self-reporting, participants report when they realize that their attention has shifted away from the ongoing task [74]. In contrast, thought probes prompt participants at specific times to report their attention states [75]. Our study uses thought probes to label changes in learner attention state because it is generally considered as an effective method [24] and, unlike self-reports, does not rely on participant meta-awareness [76].

This study explores the following questions:

- How does our model’s prediction compare to the thought probe in measuring the learner’s attention state?
- What can continuous attention monitoring reveal about the learner’s attention state? What are its implications for future designs?

### 5.1. Thought Probe and Video Material Design

Different question-and-answer thought probes have been designed, and Robison et al. [77] has compared their validity and found different thought probes to be robustly and similarly valid. Thus, we adopted probe questions from Unsworth et al. [24] and Deng et al. [78] which best match our research interest—“In the moments just prior to the probe, did you focus on the video? Yes/No”. We adopted the probe frequency and video length used in previous research [79,80], and chose a 22′20″ video on “the history of psychology” as the study material and inserted 10 probes randomly at 2-min mean intervals. At every probe, the video was paused and the question would show on screen with a black background. Following [80], participants were instructed to say “Yes” or “No” as an indication of whether their focus was on the video just prior to the probe.

### 5.2. Participants and Procedure

We recruited 24 participants (12 females, $age_{M}$ = 23.1, $age_{SD}$ = 3.72) from the university community. To minimize the impact on results from participant’s prior knowledge, we made sure that participants did not have a background in psychology and had not watched the video before. All the participants were native speakers of the language used in the video. We used the same EEG device from our data collection Section 3.1.2.

Upon arrival, participants were asked to sign a consent form and informed about the study aim. They were then asked to complete three runs of 10-min gradCPT, as per the procedure in Section 3 for model training. After a 5 min break, they were given instructions to the experiment and informed on how to respond to the probe questions [81,82]. During video learning, they were not allowed to pause, rewind the video or take notes. Throughout the experiment, participants were asked not to remove the EEG device or the electrode. The whole experiment lasted for approximately 1 h and 15 min.

## 6. Results and Discussion

### 6.1. Prediction vs. Thought Probe Result

**Figure 8:**
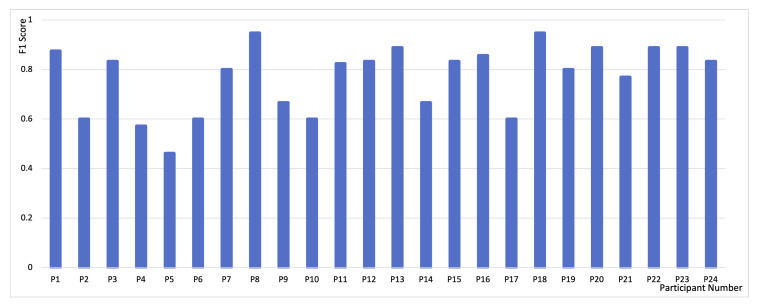
F1 score of 24 participants, P1–P24.

In total, we collected 240 probe responses. Of these, 172 were “on-task”, which is within the normal range based on previous studies [79]. The prediction accuracy was calculated based on the averaged prediction of the system 4 s prior to the probe moment. The 4-s model was chosen based on the interpretation of “just prior to the probe” in previous research [24] and the need of having odd number of labels to generate the result. The F1 score of each participant is listed in Figure 8. The overall accuracy, precision, recall, and F1 are 0.74 (SD = 0.09), 0.80 (SD = 0.16), 0.75 (SD = 0.14), 0.77 (SD = 0.14), respectively.

### 6.2. Discussion

#### 6.2.1. Comparison with Previous Studies

We compared the model performance in our video learning scenario with results from previous research on e-learning attention state monitoring. Hutt et al. [8] used eye gaze to measure mind-wandering in a computerized learning scenario, and only achieved a result of F1 = 0.59 on a 30-s time window. Huang et al. [26] measured internal thought in video learning through eye vergence behavior achieved a result of F1 = 0.74. However, their work only focused on the internal thought orientation of attention, and attention fluctuation comprises more components. Brishtel et al. [27] used a combination of EDA, eye-tracking and behavioral features to achieve a result of F1 = 0.83 on mind-wandering detection in a multimodal reading setting, though this prediction is from the level of the paragraph instead moment-to-moment. Compared with these studies, we demonstrated the usability of our technique in monitoring continuous attention fluctuation in the video learning scenario. This result has the potential of being applied to more real-world scenarios.

#### 6.2.2. Implication for Attention-Aware System in Video Learning

**Figure 9:**
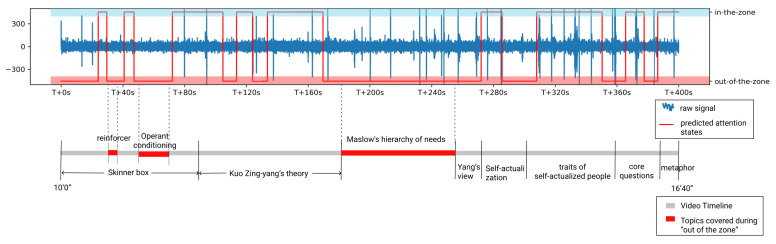
Raw EEG signal, predicted attention states, topics mentioned in the video and video timeline, all from the 400 s time segment during P8’s video viewing. The blue spikes in the raw EEG signal are eye blinks which would be removed following Section 3.2.2 before attention prediction.

Figure 9 shows the recorded EEG signal and predicted attention states of Participant 8 (P8) during video playing (10′0″–16′40″), which covers the topic from *“Skinner’s behaviorism”* to *“core questions in psychology research”*. From the figure, we can learn that the participant’s attention during video learning fluctuated quickly, even in a few seconds. This is consistent with the study done by Zhao et al. [6] where they used thought probes every 30 s in a roughly 7 min video, and caught 29% mind wandering. As previous studies trained on thought probe data cannot catch the start and the end of attention fluctuation [6,83,84], our work can serve as a first step in designing fine-grained intervention strategies to improve video learning experience and learning outcomes.

To note, despite having shown that it is feasible to measure attention fluctuation continuously, we do not envision persistent intervention in order to shift distracted or “out of the zone” users into the attentive “in the zone” state. Such persistent forms of intervention during tasks are potentially annoying to the user and unrealistic to implement. Alternative designs could account for this issue, e.g., providing reviews adaptive to learners’ attention states after they finish video viewing.

Currently, the adaptive reviews designed for attention-aware systems in the context of video learning are usually based on 4–5-min intervention scales, i.e., dividing a video into 4 min segments and providing a review for the segment with the lowest average attention level [85]. This norm could be due to the lack of precise attention measurement tools, limiting researchers to attention state summaries over a span of several minutes. However, measurements that summarize attention fluctuation over 4–5-min video segments can be insufficient in capturing attention fluctuations. Our method, in enabling measurements of continuous attention fluctuation, could provide richer data, such as the exact areas in a video where learners become distracted and move “out of the zone”. For example, in Figure 9, the shortest “out of the zone” state of P8 happened when the definition of *“reinforcer”* was introduced and lasted around 5 s. The longest “out of the zone” state of P8 happened during *“Maslow’s hierarchy of needs”* was introduced and lasted around 1 min 10 s. These data can, in turn, provide learners with an adaptive review of their learning process, i.e., they can gain insights into their conceptual learning or lack thereof. Supplementary materials, such as interactive concept maps [86], can then be created to re-emphasize concepts from which the user had initially turned their attention away. In this way, learning can be highly personalized and catered to video learners’ needs.

## 7. Overall Discussion

The validation study results suggest that our EEG-based technique achieved an average F1 score of 0.77 in attention fluctuation detection. In Section 6.2.1, through a comparison with similar works in classroom-based computerized learning, video learning, and multimodal reading scenarios, we demonstrated that our work has comparable accuracy on a smaller time-scale of 800 ms with more application potentials.

In line with the argument of Visuri et al. [10], applying a well-established psychological tool to the CS community and adapting clinical measurements of attention fluctuation to labeling would benefit the CS community in measuring attention more effectively. In our work, the use of gradCPT first increases the time resolution of attention labels, which allows us to train a classifier and measure attention fluctuation on the 800 ms time scale. Current ground truth labeling methods such as the thought probe and self-report are quite limited in this respect, as they are unable to tag massive data. Our method demonstrates the possibility to generate massive datasets of attention ground truth, which could be helpful for attention research in deep learning and emotion computing that rely on the collection of bulk data. Researchers interested in continuous attention fluctuation measurements with a longer time window can also use gradCPT by generating a single label from multiple 800 ms labels. Our experimental results suggest that it is best to take the label corresponding the largest consecutive chunk of that specific window, as it best represents the dominant attention state in that window period. For instance, for an 8-s time window with 10 labels, 0000011100, assign label 0. In summary, gradCPT provides a method to tag ground truth and generate a massive dataset for EEG-based attention research.

GradCPT, used in our experimental context, also supports the current psychological insights on the neural mechanisms behind attention control. Previous studies in psychology and neuroscience [87,88] have highlighted the importance of two brain networks, the dorsal frontoparietal attention network (DAN) and the default mode network (DMN) in mediating attention switches, and Esterman et al. [44] identified correlations between the neural and behavioral aspects of continuous attention fluctuation. For instance, the stable “in the zone” state in gradCPT was shown to be related to moderate DMN activity. On the other hand, CS researchers usually focused on different aspects of attention stimuli and defined attention within a specific context [11,89,90], e.g., the divided attention research in video learning [11] focused on only two stimuli of distraction—multitasking and environmental noise—and this potentially limits its generalization to other contexts. In our work, we distinguish attention states based on a RTV analysis in gradCPT, depending less on distractions or other environmental factors. This suggests that it could theoretically be applied to multiple domains.

In addition, the barrier for adopting our methods in future investigations is lower because it only requires a consumer-grade EEG device, which is portable, relatively low-cost, and easy to use. Real-time and fine-grained monitoring of daily attention tasks is, as a result, made accessible. Though there are debates over the validity of these devices, they focused on the validity of the derived attention index and the influence of EOG artifacts. We avoided the use of derived attention index, focused on the relatively reliable raw EEG signals, and applied the EOG removal algorithms, to ensure the validity of our work.

## 8. Challenges, Limitations and Future Work

Our work is a first step in the EEG-based classification of continuous attention fluctuation on the sub-second scale. Future research can go even further by calibrating this research to different real-world contexts, though we note that there may be several challenges in this endeavor. The first challenge pertains to the motion artifacts that could influence real-time usage in ubiquitous environments. In our video learning scenario, participants sat with minimal body movements, and were instructed to refrain from taking notes, stopping, or rewinding the video, all of which are behaviors regularly found in real-world environments. Devices similar to the Neurosky Mindwave mobile 2, a common consumer-grade EEG headband, have been used in research studying various mobile contexts [57,91], though large body movements were not usually accounted for in such research. Hence, for environments involving more body movements, additional algorithms for removing motion artifacts could be explored. Such research could expand the application potential of our technique in dynamic environments, such as for on-the-go and multitasking scenarios. To enable its use in specific real-world contexts, especially those contexts requiring highly accurate attention monitoring, e.g., driving, additional and robust noise removal approaches should be explored to remove specific noises in the environment, and context features such as wheel movement could be combined to improve classification accuracy. The second challenge concerns artifact removal. In the beginning stage, we identified the influence of EOG on the EEG signals, thus we removed EOG signal noise via feature replacement. However, previous studies showed that spontaneous eye blink rate [92] as well as frontalis muscle activity [93] can be used as an index of attention. Combining additional measurements such as EOG and EMG with EEG could improve classification accuracy and provide insights into attention states.

The limitations of our research concerns the accuracy of model training and the computation cost of DWT when applied online. First, we used a commercial EEG device with the measuring electrode on Fp1 because of its common usage and general applicability. The accuracy of our results is thus limited to the consumer-grade tool and its single-channel EEG input option. In addition, the model is trained in a person-specific manner, i.e., we record the user’s gradCPT performance prior to use as a means of calibration. While it is undesirable from the usability point of view, our model requires fewer data points due to the small window size, thus methods such as transfer learning [94] could significantly reduce the duration of the gradCPT to make for easier calibration in the future. Second, DWT is computationally intensive. Considering that sensor computing hardware is becoming increasingly accessible, designing hardware optimized for DWT computation, as suggested by Elsayed et al. [95], could allow for efficient implementation when serving online.

## 9. Conclusions

In this paper, we proposed a technique to measure moment-to-moment continuous attention fluctuation with the consumer-grade EEG device. To achieve this, we first applied the gradCPT, a well-established model in psychology to collect the ground truth of attention fluctuation. The gradCPT is able to precisely label attention state at the sub-second time scale of 800 ms, and has high potential for wide and general usage. Through collecting EEG signals with a consumer-grade device, we trained a classifier and achieved an accuracy of 73.49% in attention fluctuation detection. We then tested its generalization in a video learning scenario and achieved an average F1 score of 0.77 in attention fluctuation prediction. This result is comparable with previous studies on attention in e-learning scenarios, but contributes further by offering measurements at this level of accuracy on the sub-second scale, and is usable in contexts beyond e-learning. Overall, our work demonstrates the feasibility of using a commercial EEG device to measure moment-to-moment continuous attention fluctuation, which has broad potential use in building attention-aware systems and future attention related research. 

## Figures and Tables

**Table 1:** Summary of the recent works in attention state classification using automatic physiological data.

Sensors	Attention States	Attention State Labeling Method	Time Scale of Ground Truth	Classifier	Result
Thermal image and Eye tracking [12]	Sustained attention	Controlled tasks	3 min each task	Logistic Regression	75.7% AUC score for user-independent condition-independent
	Alternating attention				87% AUC score for user-independent condition independent
	Selective attention				77.4% AUC score for user-dependent
	Divided attention				
EDA [34]	Engaged	Self-report questionnaires	45 min each questionnaire (after a lecture)	SVM	0.60 for accuracy
	Not engaged				
PPG [11]	Full Attention (FA)	Designed tasks based on the combination of internal and external distractions	8 min each task	RBF-SVM classifiers	50% for FA vs. EDA vs. LIDA vs. HIDA
	Low internal divided attention (LIDA)				72.2% for FA vs. EDA
	High internal divided attention (HIDA)				75.0% for FA vs. LIDA
	External divided attention (EDA)				83.3% for FA vs. HIDA

**Table 2:** Features computed for theta/alpha/beta/gamma/delta band and descriptions.

Feature	Description
Approx. Entropy	Approximate entropy of the signal
Total variation	Sum of gradients in the signal
Standard variation	Standard deviation of the signal
Energy	Sum of squares of the signal
Skewness	Sample skewness of the signal

## Data Availability

The EEG data used to support the findings of this study are from NUS-HCI Lab, National University of Singapore. To protect subject privacy, data sharing is restricted based on request from the corresponding author and for researchers who meet the criteria for access to confidential data only.

## Abbreviations

The following abbreviations are used in this manuscript:

CNN	Convolutional neural network
CPT	Continuous performance test
CV	Cross-validation
DWT	Discrete wavelet transform
EDA	Electrodermal activity
EEG	Electroencephalography
EMG	Electromyogram
EOG	Electrooculogram
ER	Error rate
fMRI	Functional magnetic resonance imaging
gradCPT	Gradual onset CPT
iEEG	Intracranial electroencephalography
kNN	k-nearest neighbor
LSTM	Long short term memory
PPG	Photoplethysmogram
RT	Response time
RTV	Response time variability
SART	Sustained-attention-to-response task
SVM	Support Vector Machine
VTC	Variance Time Course
